# Editorial: Vetinformatics: an insight for decoding livestock systems through *in silico* biology, volume II

**DOI:** 10.3389/fvets.2025.1672786

**Published:** 2025-08-13

**Authors:** Jun-Mo Kim, Rajesh Kumar Pathak

**Affiliations:** ^1^Department of Animal Science and Technology, Chung-Ang University, Anseong-si, Republic of Korea; ^2^Pere Virgili Institute for Health Research (IISPV), Tarragona, Spain

**Keywords:** vetinformatics, veterinary science, animal health, machine learning, One Health, multi-omics, molecular docking and molecular dynamics (MD) simulation, vaccine design

Global demand for food is increasing alongside the rapidly growing human population, which is projected to reach nearly 10 billion by 2050 (1). Livestock systems face unprecedented pressures as emerging pathogens increase because of climate change, antimicrobial resistance, and the urgent need for sustainable intensification (2). Traditional veterinary science approaches have struggled to address these multidimensional challenges. Vetinformatics is an emerging interdisciplinary field poised to revolutionize animal health and production by decoding the complex molecular mechanisms governing livestock biology (3). Vetinformatics integrates computational biology, data science, and multiomics technologies into core veterinary disciplines, such as pharmacology, genetics, and epidemiology (2). This represents a new bioinformatic research concept that employs an interdisciplinary approach to understand the complex molecular mechanisms of animal systems. This synergy accelerates research, ensures food security, optimizes animal welfare, and support One Health (2, 4).

The catalyst for vetinformatics is the deluge of data from next-generation sequencing and functional genomics (3, 5). Genome assemblies for cattle, pigs, poultry, and other livestock provide blueprints for identifying disease resistance genes, markers, and metabolic pathways. For example, transcriptomics reveals how genes regulate reproductive efficiency, whereas metagenomics reveals how rumen microbiomes influence feed efficiency in livestock (2). However, the raw data alone are inert. Vetinformatics transforms them into actionable knowledge through tools, such as SPAdes, for genome assembly, edgeR for RNA-Seq data analysis, and GATK for variant detection, enabling species-specific insights (2). Additionally, predictive modeling, such as molecular dynamics simulations, assesses how mutations affect protein stability, whereas systems biology integrates genomics, proteomics, and metabolomics to model host-pathogen interactions. Furthermore, molecular docking and virtual screening of compound libraries against target receptors have accelerated veterinary medicine research (3).

These implications extend beyond productivity alone. Vetinformatics underpins vaccine design and offers alternatives to antibiotics (2). Machine learning algorithms classify livestock behavior from video data (6), thereby enabling early disease detection. However, challenges remain, such as non-integrated data, scarce species-specific databases, and training gaps between veterinarians and computational biologists (3). As we stand at these crossroads, vetinformatics is not merely an adjunct to veterinary science; it is its evolving backbone. Bridging genotype to phenotype equips us to foster resilient livestock systems, mitigate zoonotic risks, and ethically meet global food needs. The future will demand nothing less than a computationally empowered revolution in animal health (7).

“*Vetinformatics: an insight for decoding livestock systems through in silico biology, volume II*” attracted eight submissions, with six high-quality contributions accepted for this Research Topic.

The first article in this Research Topic, titled “*Machine learning-based risk prediction model for canine myxomatous mitral valve disease using electronic health record data*,” analyzed the records of 143 MMVD-affected dogs (2018–2022). Researchers tested four machine learning models to predict the risk of heart failure. The random forest model emerged as the most effective (88% accuracy), with heart imaging and radiograph measurements ranking as the top predictors. Packed cell volume, respiratory rate, and chloride levels also showed strong predictive values. This tool helps veterinarians make more informed prognostic decisions for dogs with this common heart condition (Kim et al.). The second article, titled “*Aflatoxin contamination of animal feeds and its predictors among dairy farms in Northwest Ethiopia: One Health approach implications*,” highlights the widespread contamination of dairy feeds with aflatoxins in this region. This study identified poor storage practices, certain feed ingredients, and seasonal factors as major contributors. These findings point to a serious risk to milk safety and underline the need for better awareness and health-based interventions at the farm level (Tadele et al.). The third article, entitled “*Structure-based virtual screening and molecular dynamics studies to explore potential natural inhibitors against 3C protease of foot-and-mouth disease virus*,” used molecular modeling to search thousands of natural compounds targeting a key viral protein. Researchers identified promising candidates with strong binding affinities. These findings open the door for the development of new antiviral drugs for foot-and-mouth disease in livestock (Sahoo et al.). The fourth article, titled “*RNA sequencing analysis of sexual dimorphism in Japanese quail*,” shows that quails and chickens share similar sex-related gene expression patterns. Similar to chickens, quails lack global Z-chromosome dosage compensation. Some genes shift sex bias by tissue or stage, offering insights into avian sexual dimorphism and supporting future genetic research (Park et al.). The fifth article, titled “*Identifying feature genes of chickens with different feather pecking tendencies based on three machine learning algorithms and WGCNA*,” analyzes gene expression in chickens with varying feather-pecking behaviors. By integrating network analysis and three machine learning approaches, this study identified five key genes—*NUFIP2, ST14, OVM, GLULD1*, and *LOC424943*—which were validated using ROC analysis in an independent dataset. These findings provide a foundation for further research on the behavioral traits of poultry (Wen et al.). Concluding this Research Topic is an article titled “*Genome-wide association studies with prolapsed gland of the third eyelid in dogs*.” This study conducted genome-wide association analyses of 653 brachycephalic dogs and 788 dogs with various head shapes, identifying significant SNPs on chromosomes CFA3 and CFA22. Notably, genes, such as *NR2F1, DIO3*, and *TTC8*, are associated with eye development and disease. These findings offer new insights into the genetic underpinnings of cherry eye disease in dogs (Zeng et al.).

To address the increasing need for computational resources in veterinary science, this Research Topic highlights the advances and potential of vetinformatics. It explores how these approaches can help unravel complex livestock biology and offer practical ways to enhance animal health and welfare.
